# A novel prognostic gene set for colon adenocarcinoma relative to the tumor microenvironment, chemotherapy, and immune therapy

**DOI:** 10.3389/fgene.2022.975404

**Published:** 2023-01-09

**Authors:** Hui Zhou, Yongxiang Wang, Zijian Zhang, Li Xiong, Zhongtao Liu, Yu Wen

**Affiliations:** Department of General Surgery, Second Xiangya Hospital, Central South University, Changsha, China

**Keywords:** TMB, immune, prognosis, chemotherapy, drug sensitivity, colon adenocarcinoma

## Abstract

**Background:** Colon adenocarcinoma (COAD) is a common aggressive malignant tumor. Heterogeneity in tumorigenesis and therapy response leads to an unsatisfactory overall survival of colon adenocarcinoma patients. Our study aimed to identify tools for a better prediction of colon adenocarcinoma prognosis, bolstering the development of a better personalized treatment and management.

**Method:** We used the least absolute shrinkage and selection operator (LASSO) Cox model to analyze the prognosis-related gene datasets from the Gene Expression Omnibus (GEO) database and verified them using The Cancer Genome Atlas (TCGA) database. The area under the curve (AUC) was calculated using the receiver operating characteristic (ROC) curve to evaluate the predictive ability of the risk score model. Gene Set Enrichment Analysis (GSEA) was used to identify the significantly enriched and depleted biological processes. The tumor immune dysfunction and exclusion (TIDE) algorithm was taken to explore the relationship between the risk score and immunotherapy. The observations collectively helped us construct a nomogram to predict prognosis. Finally, the correlation between drug sensitivity and prognostic gene sets was conducted based on the Cancer Therapeutics Response Portal (CTRP) analyses.

**Results:** We constructed a scoring model to assess the significance of the prognosis risk-related gene signatures, which was relative to common tumor characteristics and tumor mutational burdens. Patients with a high-risk score had higher tumor stage and poor prognosis (*p*< 0.05). Moreover, the expressions of these genes were in correlation with changes in the tumor microenvironment (TME). The risk score is an independent prognostic factor for COAD (*p*< 0.05). The accuracy of the novel nomogram model with a risk score and TNM-stage prediction prognosis in the predicting prognosis was higher than that of the TNM stage. Further analysis showed that a high-risk score was associated with tumor immune rejection. Patients with a low-risk score have a better prognosis with chemotherapy than those with a high-risk score. Compared to patients in the high-risk group, patients in the low-risk group had a significant survival advantage after receiving chemotherapy. In addition, the prognostic gene sets aid the assessment of drug sensitivity.

**Conclusion:** This study establishes a new prognostic model to better predict the clinical outcome and TME characteristics of colon adenocarcinoma. We believe, our model also serves as a useful clinical tool to strengthen the functioning of chemotherapy, immunotherapy, and targeted drugs.

## Introduction

Colon adenocarcinoma (COAD) is the main pathological type of colon cancer and the second leading cause of cancer deaths worldwide (Yoshino et al., 2018; Keum and Giovannucci, 2019). Approximately 900,000 COAD patients die each year from this malignancy due to its late clinical diagnosis (Dekker et al., 2019). Moreover, the incidence and mortality rates of COAD have been continuously growing, owing to the unsatisfactory prognosis of advanced COAD cases. The poor prognosis of COAD may also be due to its tumor recurrence and metastasis, characteristic of the disease. The 5-year and 10-year survival rates of most patients with metastatic COAD are 40% and 20%, respectively (Zhou et al., 2022). Treatment decisions are primarily based on assessing the tumor node metastasis (TNM) staging system (Amin et al., 2017). COAD is a heterogeneous cancer with genetic and clinicopathologic features regulating its occurrence and development (Gu et al., 2020). However, TNM staging fails to reveal its biological heterogeneity (Zhou et al., 2021). Moreover, an accurate prediction of the survival duration of COAD patients is helpful for clinical decision-making, warranting an urgent need to find more precise prognosis-predictive tools.

Currently, the standard treatment modalities for patients with COAD include surgery, adjuvant chemotherapy, and radiotherapy. It is challenging to remove all the cancer cells *via* surgery, causing advanced COAD patients to receive further treatment with adjuvant chemotherapy and radiotherapy (Ganesh et al., 2019). Chemotherapeutic drugs are non-specific and cytotoxic in nature with many side effects to any normal growing and dividing cell of the body. Notably, immunotherapy is one of the novel and current alternative treatments for COAD patients. Immune checkpoint therapy, which received a regulatory approval in 2017, primarily treats severely mutated COAD patients with deficient mismatch repair (dMMR) or high levels of microsatellite instability (MSI-H) (Picard et al., 2020). However, COAD patients, upon receiving adjuvant immunotherapy, may exhibit an immune exclusion response (Fan et al., 2021). Moreover, different chemotherapy drugs elicit variable prognoses for different types of COAD patients. However, choosing a personalized treatment plan still remains challenging and confusing. Hence, the need of the hour is to identify a prognostic model to predict the survival outcomes of COAD patients. The aim was to use this model to clinically guide COAD treatment decisions.

In this study, large data from a cohort of COAD patients from TCGA database were screened for differentially expressed prognostic risk-associated genes. These genes were chosen from the GEO database and verified using TCGA expression data. Herein, we aimed to construct a novel prognostic risk scoring method for COAD that could lead to the administration of a better personalized treatment and management.

## Materials and methods

### Data collection and preprocessing

The expression profiles were downloaded from two platforms: the GSE39582 dataset from the Gene Expression Omnibus (GEO) database (https://www.ncbi.nlm.nih.gov/geo/), and transcriptome profiling (TCGA-COAD-RNAseq) and single-nucleotide variant (TCGA-COAD-SNV) datasets from The Cancer Genome Atlas (TCGA) database (https://www.tcga.org). Single-nucleotide variant (SNV) datasets from TCGA. TCGA-COAD-RNAseq contains 515 samples, including 473 tumor tissue samples and 41 normal solid tissue samples. TCGA-COAD-SNV contains 896 samples, including 448 tumor tissue samples and 448 normal samples. GSE39582 contains 585 samples, containing 566 tumor tissue samples and 19 normal tissue samples. We carried out quantile normalization for expression profiles with the preprocessCore package. Then, we carried out survival analysis and univariate Cox regression analysis for every gene in GSE39582 to obtain the overlap genes as prognostic genes (with the cutoff *p*-value<0.05) with survival packages. Using the Human Protein Atlas (HPA) database (https://www.proteinatlas.org), by immunohistochemical (IHC) staining, we tested normal intestinal tissue and performed prognosis in COAD organization gene expression differences in the protein level (Asplund et al., 2012).

### Construction and external validation of the risk-scoring model

We took GSE39582 as the training dataset to construct a risk-scoring model based on these prognostic genes; the robust prognosis risk-related genes were selected from all prognostic genes *via* a risk score evaluated by the LASSO regression model. To validate the effect of predictive ability of the robust prognosis risk-related genes, the ROC curve was applied to calculate the area under the curve (AUC) on the foundation of the risk score model. The risk-scoring model obtained from GSE39582 was validated by TCGA data *via* Kaplan–Meier survival analysis and ROC curves. Taking the median risk score as the cut-off point, the survival analysis was carried out.

### Comprehensive analysis about prognostic gene sets

To analyze the biological process based on the risk score group, we carried out Gene Set Enrichment Analysis (GSEA) with the clusterProfiler package. Seven gene sets (GO_ACTIVATION_OF_IMMUNE_RESPONSE, GO_IMMUNE_RESPONSE_TO_TUMOR_CELL, GO_MACROPHAGE_ACTIVATION_INVOLVED_IN_IMMUNE_RESPONSE,GO_NATURAL_KILLER_CELL_MEDIATED_IMMUNE_RESPONSE_TO_TUMOR_CELL, GO_POSITIVE_REGULATION_OF_CYTOKINE_PRODUCTION_INVOLVED_IN_IMMUNE_RESPONSE, GO_POSITIVE_REGULATION_OF_NATURAL_KILLER_CELL_MEDIATED_IMMUNE_RESPONSE_TO_TUMOR_CELL, and GO_T_CELL_MEDIATED_IMMUNE_RESPONSE_TO_TUMOR_CELL) were obtained from GSEA (http://www.gsea-msigdb.org/gsea/index.jsp). Moreover, 14 gene sets (angiogenesis, apoptosis, cell cycle, differentiation, DNA damage, DNA repair, EMT, hypoxia, inflammation, invasion, metastasis, proliferation, quiescence, and stemness) were obtained from CancerSEA (http://biocc.hrbmu.edu.cn/CancerSEA/). We performed a gene set variation analysis about immune signatures and tumor signatures and analyzed the relationship between the risk score and GSVA score. Also, we analyzed immune infiltration with different tools to know about the status of immune filtration in different risk groups. Combining the clinical information, we explored the difference between high-risk and low-risk groups in the TNM stage and drug reaction. Apart from these, we combined the TCGA-COAD-RNAseq dataset and the TCGA-COAD-SNV dataset to analyze the genetic background behind the two groups with the maftools package.

### The clinical value analysis of prognostic gene sets

We investigated the therapeutic value of genes associated with a robust prognostic risk. The tumor immune dysfunction and exclusion (TIDE) algorithm was used to explore the relationship between the risk score and immunotherapy. We also analyzed the relationship between the risk score and chemotherapy. Moreover, we combined the risk score and TNM stage to construct a novel nomogram model with the rms package to improve the model value in predicting prognosis.

### Drug sensitivity data analysis

We collected the corresponding mRNA gene expression from the genomics of the Cancer Therapeutics Response Portal (CTRP) and merged the mRNA expression and drug sensitivity data. Pearson correlation analysis was performed to obtain the correlation between mRNA expression and drug IC_50_ values. An FDR-adjusted *p*-value was used in all the analyses (Liu et al., 2018).

## Results

### Construction and validation of the prognostic model

**FIGURE 1:**
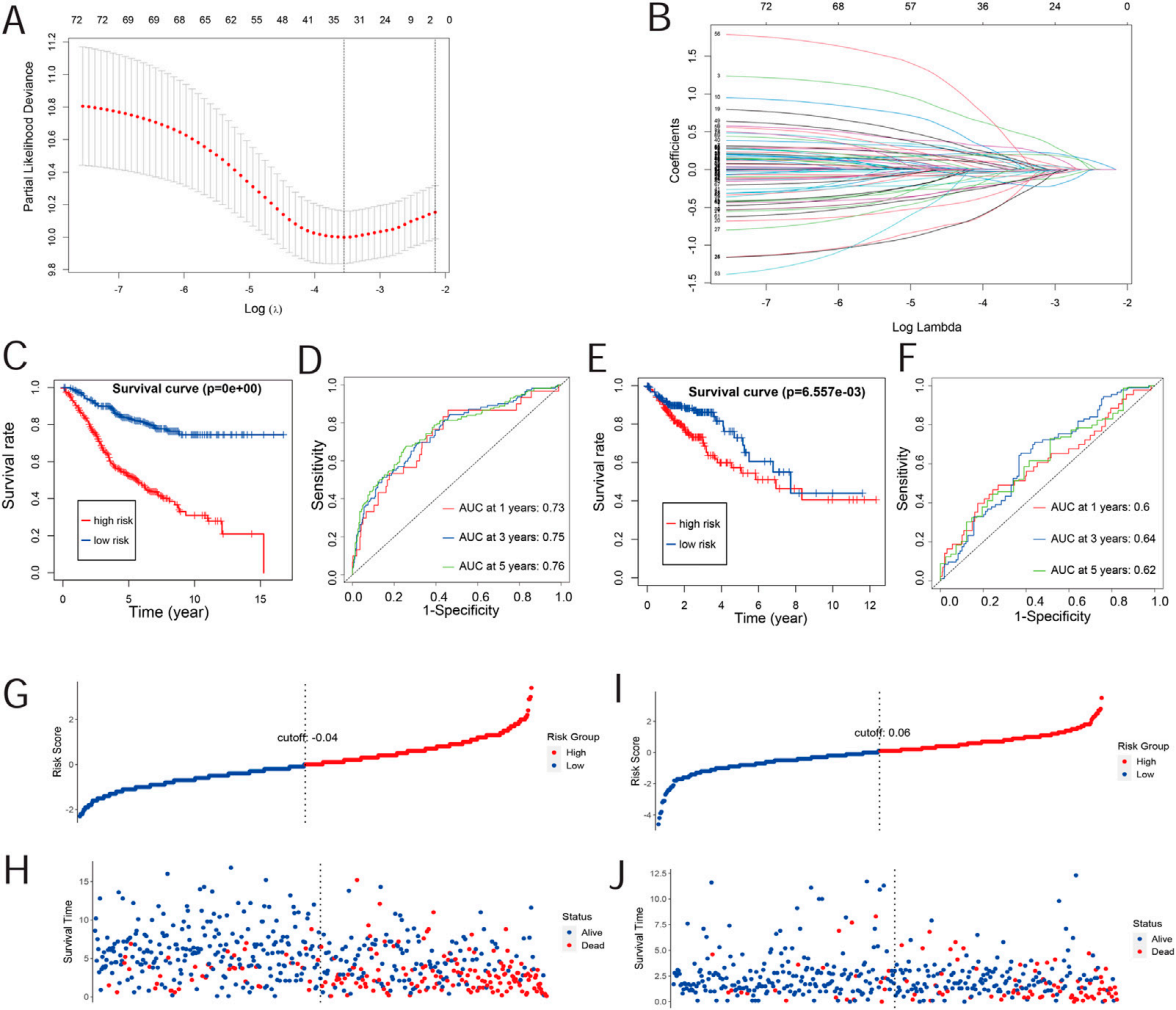
Risk-scoring model construction and validation. **(A)** Illustration for the LASSO coefficient spectrum of prognostic genes. **(B)** Adjusted parameters of the LASSO regression model. **(C)** Kaplan–Meier curve analysis of OS in high-risk and low-risk groups based on the GEO database. **(D)** Kaplan–Meier curve analysis of OS in high-risk and low-risk groups based on the TCGA database. **(E)** ROC curve of risk scores in the GEO database. **(F)** ROC curve of risk scores in TCGA database. **(G,H)** Overview of the survival time and distribution of risk scores of the GEO database. **(I,J)** Overview of the survival time and distribution of risk scores of TCGA database.

Survival analyses helped obtain the prognosis-related gene expression profile for COAD patients. Moreover, Cox regression analysis in the GEO dataset, which was verified by TCGA dataset, also helped in the process. The analysis identified a total of 76 prognosis-related genes. In the LASSO regression model, 33 genes were identified as robust prognosis risk-related genes (Figures 1A, B). The 33 genes selected for the model included *ATOH1*, *C4orf47*, *CPA4*, *DNASE1L1*, *ERFE*, *F2RL2*, *FBXO39*, *FZD3*, *HPCAL4*, *ICOS*, *INHBB*, *ITLN1*, *KIF7*, *KLHL26*, *LINC00629*, *LRRC29*, *MMP12*, *MYL6B*, *NPM3*, *PCBD1*, *PLEC*, *POLR2F*, *POU5F1P4*, *PRRX2*, *PTPRU*, *PTTG3P*, *RNF112*, *SERPINB7*, *SLCO1A2*, *TH*, *TMEM39B*, *TRDV3*, and *ZDHHC1*. The detailed characteristics of these prognostic genes in this study are given in Supplementary Table S1. Most prognostic genes were differentially expressed between COAD and normal tissues (Supplementary Figure S1). *ATOH1*, *HPCAL4*, *ITLN1*, *POLR2F*, *RNF112*, *SERPINB7*, *SLCO1A2*, *TH*, and *TMEM39B* were significantly downregulated in COAD tissues (*p* < 0.05) compared to those in normal tissues. *C4orf47*, *CPA4*, *DNASE1L1*, *ERFE*, *F2RL2*, *FBXO39*, *FZD3*, *INHBB*, *KLHL26*, *LRRC29*, *MMP12*, *MYL6B*, *NPM3*, *PCBD1*, *POU5F1P4*, *PRRX2*, *PTPRU*, *PTTG3P*, *TRDV3*, and *ZDHHC1* were significantly upregulated in COAD samples (*p* < 0.05) compared to those in normal tissues. Here, we also used the Human Protein Atlas (HPA) database to validate the expression of these prognostic genes at the protein level (Supplementary Figure S2).

The risk score was evaluated by the coefficient of each of these genes. The formula for the risk score model is as follows: $risk\;score=\overset{n}{\underset{i=1}{\sum }}coefi*expri$ . The median risk score was the cut-off point for dividing the patients. The Kaplan–Meier survival analysis showed that the OS in the low-risk group was significantly higher than that in the high-risk group (*p* < 0.0001, Figure 1C) in the GEO datasets. The ROC curve showed that the AUC values of the 1-, 3-, and 5-year OS were 0.73, 0.75, and 0.76, respectively, in the GEO dataset (Figure 1D). Moreover, TCGA datasets were used for further validation; the OS in the low-risk group was significantly higher than that in the high-risk group (*p* < 0.0001, Figure 1E). The ROC curve indicated that the AUC values of the 1-, 3-, and 5-year OS were 0.6, 0.64, and 0.62, respectively (Figure 1F). Moreover, the low-risk score group has a better outcome of prognosis (Figures 1G, H). Similarly, the risk score group also showed a better outcome (Figures 1I, J) in the validation cohort. The heatmap depicts the expression pattern of prognosis risk-related genes between the high- and low-risk groups in the training and validation cohorts (Supplementary Figure S3). These results collectively indicated that these 33 genes, making up a prognostic gene set, can be used to construct a novel risk model to accurately predict the prognosis of COAD patients.

### Roles of the prognostic gene sets in regulating the tumor immune microenvironment and tumor signatures

**FIGURE 2:**
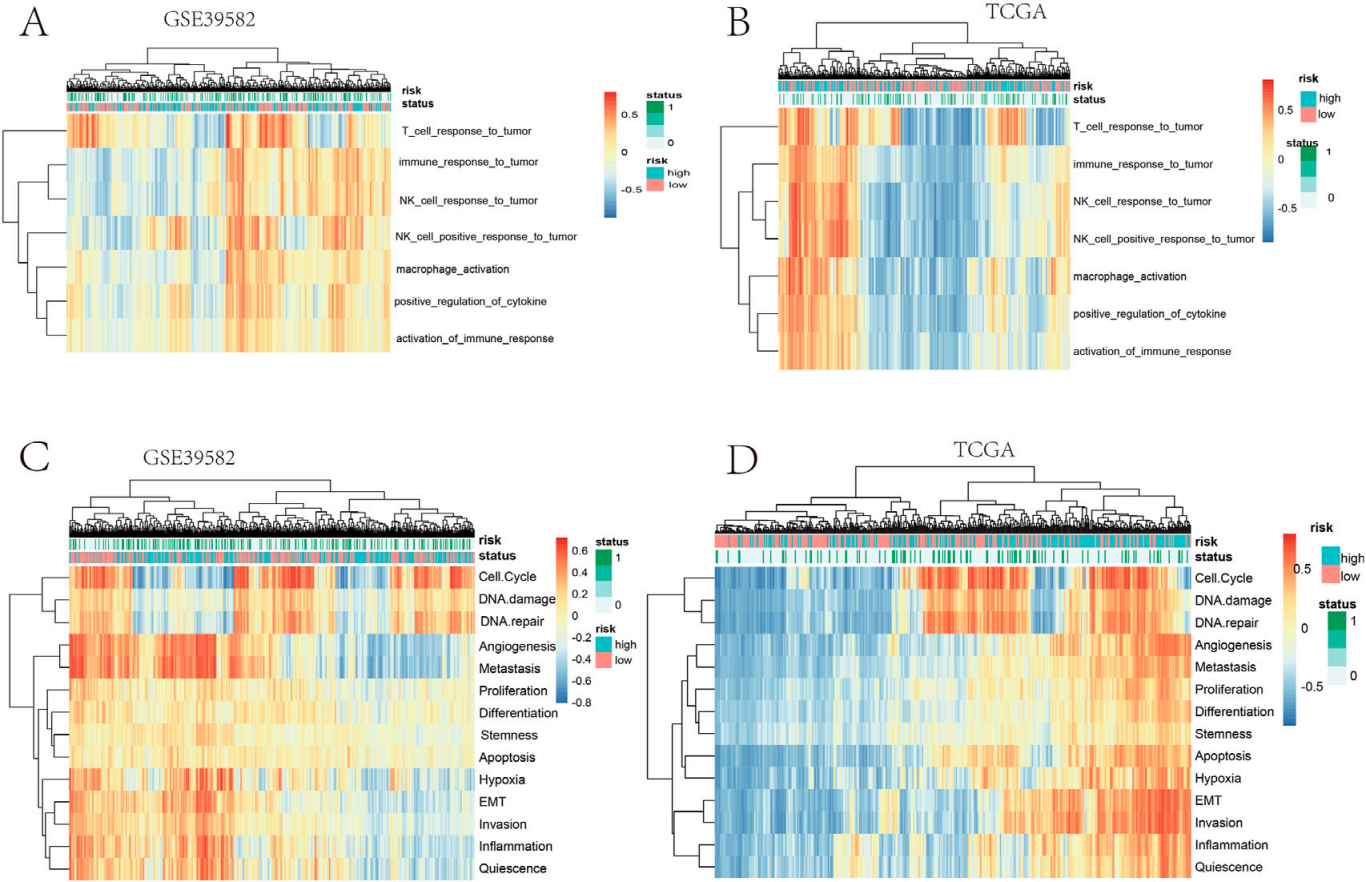
**(A,B)** Heatmap clustering of the risk score and immune signatures in the training cohort. **(C,D)** Heatmap clustering of the risk score and tumor signatures in the validation cohort.

**FIGURE 3:**
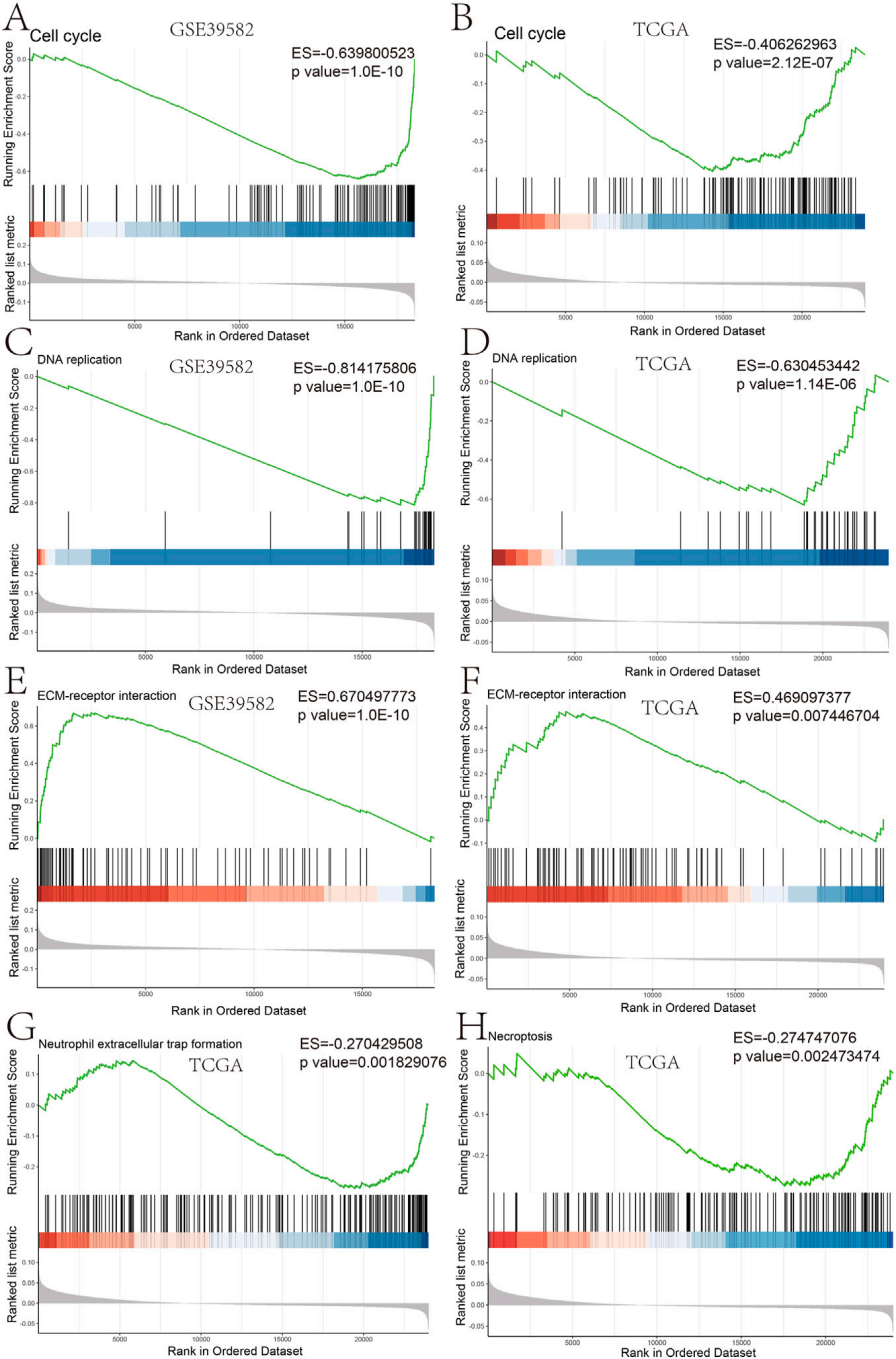
GSEA plot of the biological process based on the risk score GSEA analysis in GSE39582 **(A,C,E)** and GSEA analysis in TCGA **(B,D,F–H)**.

**FIGURE 4:**
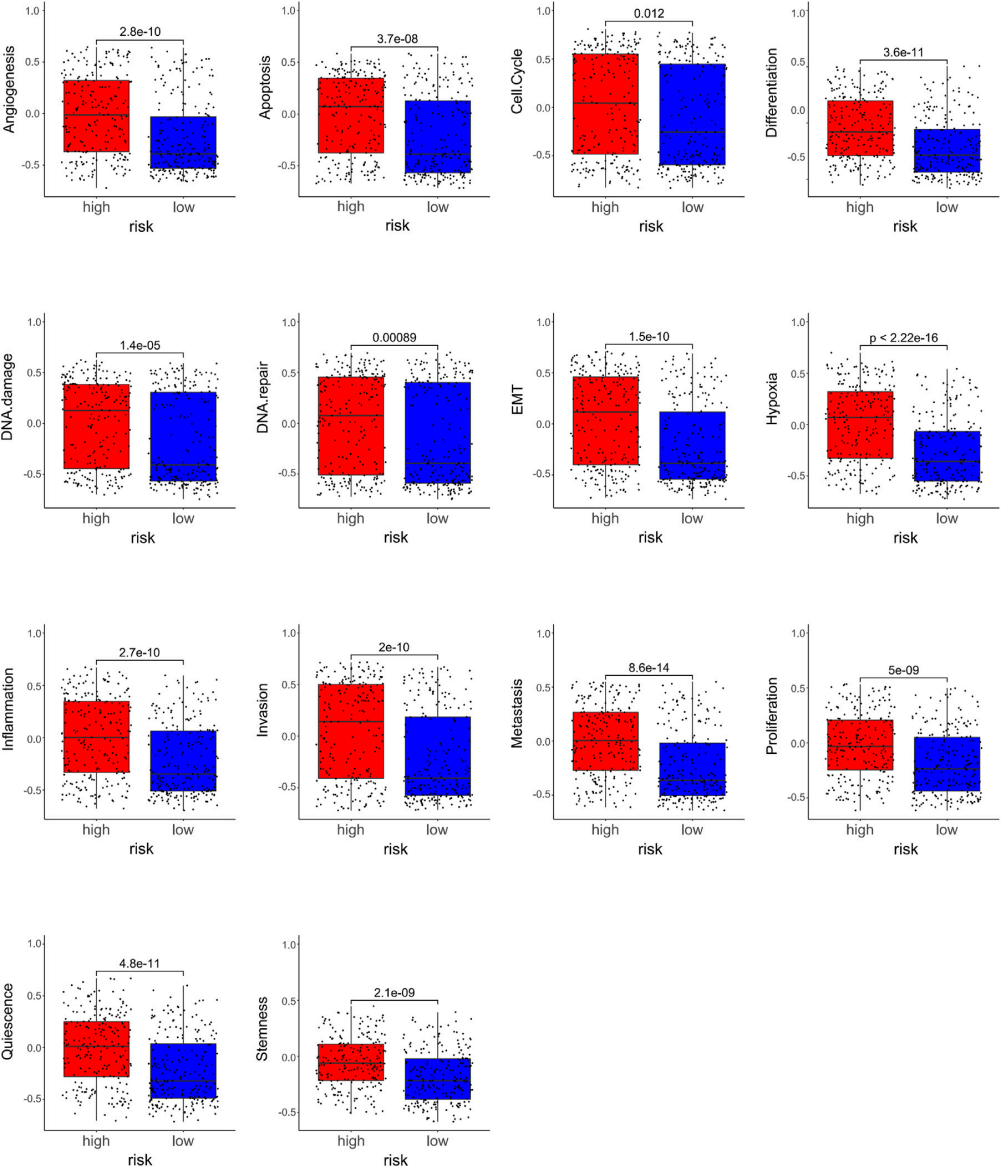
Relationships between the compositions of the risk scores and the tumor signatures.

Furthermore, we studied the relationships between the prognostic gene sets, tumor immune microenvironment, and tumor signatures. The heatmap in Figures 2A, B shows the proportions of tumor-infiltrating natural killer (NK) cells, T cells, neutrophils, and macrophages in the TME. It also indicates that the immune response to the tumor corroborated our prognostic risk score (*p* <0.05). Moreover, the heatmap in Figures 2C, D shows that the proportions of the cell cycle, DNA damage, DNA repair, angiogenesis, metastasis, proliferation, differentiation, stemness, apoptosis, hypoxia, EMT, invasion, inflammation, and quiescence are significantly related to our prognostic risk score (*p* < 0.05). Also, we carried out a GSEA to analyze the enriched biological processes based on the risk score group. The GSEA showed enrichment of the GO biological processes like cell cycle (ES = −0.406262963; *p* = 2.12225E-07), DNA replication (ES = −0.630453442; *p* = 1.14446E-06), ECM–receptor interaction (ES = 0.469097377; *p* = 0.007446704), neutrophil extracellular trap formation (ES = −0.270429508; *p* = 0.001829776), and necroptosis (ES = −0.274747076; *p* = 0.002473474) in TCGA dataset when comparing the high-risk group with the low-risk group (Figure 3). GSEA of the GSE39582 dataset revealed that a higher risk score was closely related to the enrichment of gene sets related to the cell cycle (SE = −0.639800523; *p* = 1E-10), DNA replication (SE = −0.814175806; *p* = 1E-10), and ECM–receptor interactions (ES = 0.670497773; *p* = 1E-10) (Figure 3). The risk score is closely correlated with tumor signatures, including cell cycle, DNA damage, DNA repair, angiogenesis, metastasis, proliferation, differentiation, stemness, apoptosis, hypoxia, EMT, invasion, inflammation, and quiescence (Figure 4). A *p*-value cut-off of <0.05 revealed that the high-risk score group had a higher GSVA score in the aforementioned 14 tumor signatures.

### Correlation of a mutation landscape within the prognostic gene sets

**FIGURE 5:**
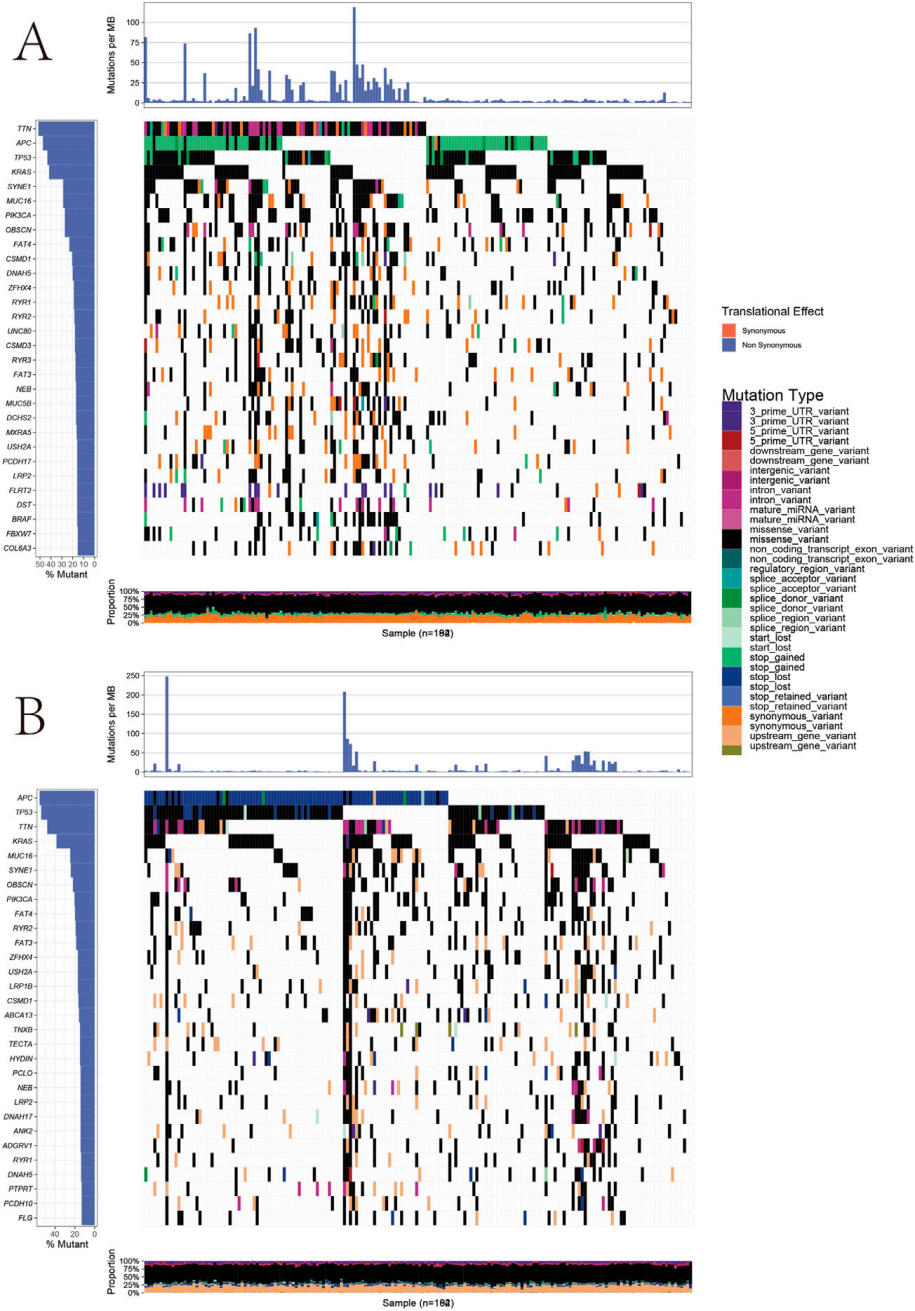
Landscape of mutations between high-risk groups and low-risk groups. **(A)** Heatmap illustrates the co-occurrence and mutually exclusive mutations in high-risk groups. **(B)** Heatmap illustrates the co-occurrence and mutually exclusive mutations in low-risk groups.

**FIGURE 6:**
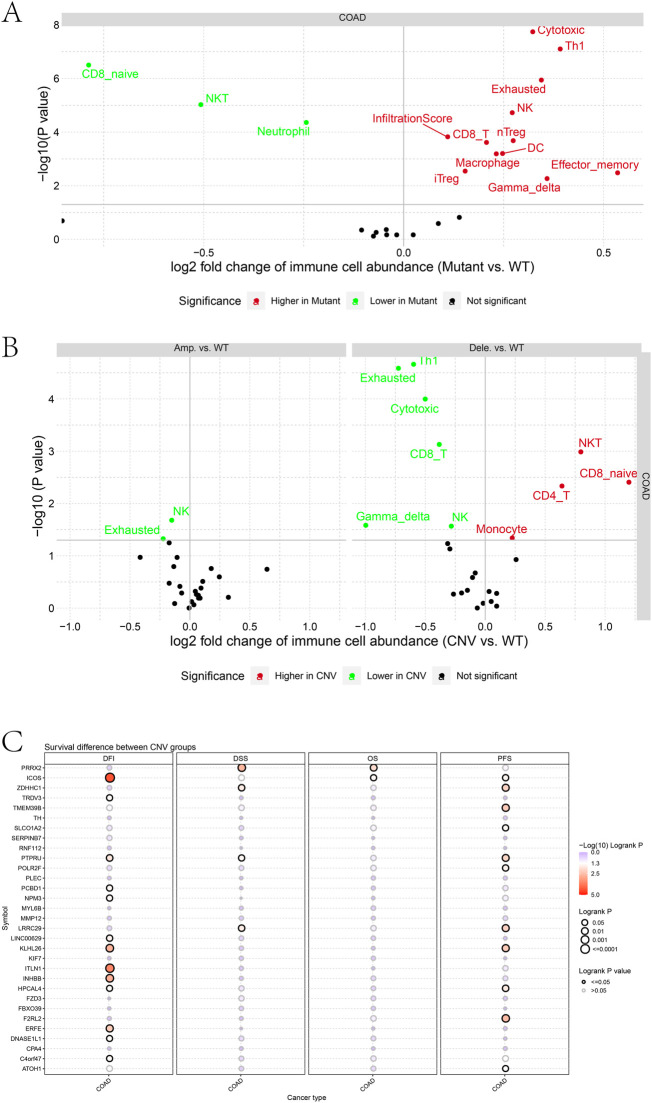
**(A)** Differences in immune cell abundance between mutant and WT groups. **(B)** Differences in immune cell abundance between CNV and WT groups. **(C)** Survival difference between CNV groups.

We further analyzed the tumor mutational burden between the high- and low-risk groups. Figures 5A, B show how the mutation frequency and mutation spectrum of the mutated genes are higher in the high-risk group. *TTN* was the most significantly mutated gene in the high-risk group, while *APC* was the most significantly mutated gene in the low-risk group. Supplementary Figure S4A shows that the co-occurrence and mutually exclusive mutations were investigated and were observed in the high- and low-risk groups. In the high-risk group, *SYNE1*, *MUC16*, *OBSCN*, and *DNAH5* mutations almost co-occurred with *TTN* mutations (*p* < 0.01), while *ZFHX4* co-mutated with *OBSCN*, *FAT4*, *MUC16*, and *DNAH5* (*p* < 0.01). Moreover, *TP53* and *MUC16* mutations were almost mutually exclusive in the low-risk group (*p* < 0.01), which had a higher tumor mutational burden (TMB) than the low-risk group (*p* = 0.041) (Supplementary Figure S4B). In addition, *BRAF* mutations showed higher scores in the prognostic gene sets than the wild-type mutations (*p* = 0.011) (Supplementary Figure S4C). Moreover, macrophages, NK cells, DC cells, and CD8+T cells were increased in the mutant type, compared to the wild type, while the natural killer T cells (NKT), neutrophils, and naive CD8^+^ T cells decreased (Figure 6A). Moreover, the immune cells in the mutant were utterly exhausted. The genome rearrangement-driven copy number variation (CNV) generally refers to an increase or decrease in the copy number of a large genome segment, usually more than 1 KB in length (Lye and Purugganan, 2019). The number of NK cells in the mutant group was significantly reduced compared to the wild-type group (Figure 6B). When the copy number decreases, CD8 T cells, NK cells, and Th1 cells decrease, while NKT cells and CD4 T cells increase.

Furthermore, differences in prognostic gene sets regarding tumor copy number variation and patient prognosis were also investigated. CNVs in *ICOS*, *TRDV3*, *PTPRU*, *PCBD1*, *NPM3*, *LINC00629*, *KLHL26*, *ITLN1*, *INHBB*, *HPCAL4*, *ERFE*, *DNASE1L1*, and *C4orf47* were associated with a disease-free interval (DFI). Patients may change the disease-free survival (DFS) when *PRRX2*, *ZDHHC1*, *PTPRU*, and *LRRC29* have copy number variations. In addition, CNVs in *PRRX2* and *ICOS* change the OS of patients. CNVs in *ICOS*, *ZDHHC1*, *TMEM39B*, *PTPRU*, *POLR2F*, *LRRC29*, *KLHL26*, *HPCAL4*, *F2RL2*, and *ATOH1* genes were associated with a progression-free survival.

### Correlation of clinical features with prognostic gene sets

**FIGURE 7:**
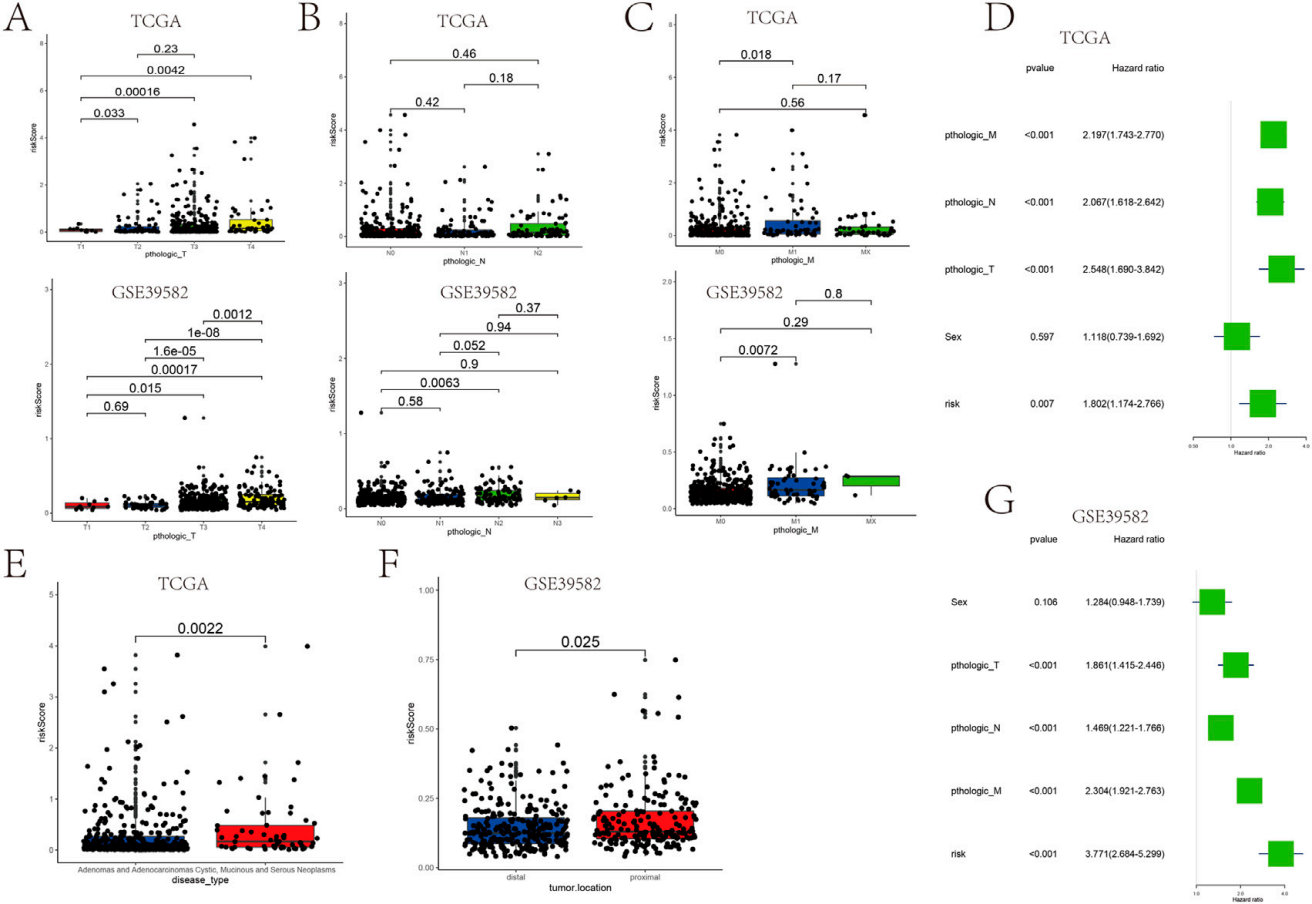
Association between the risk score and the patient’s clinical characteristics. **(A–C)** Association between the tumor size (T), tumor node (N), tumor metastasis (M), and risk score in TCGA cohort and GSE39582. **(D)** Univariate analysis of factors influencing patient prognosis in TCGA cohort. **(G)** Multifactorial analysis of factors influencing GSE39582 patient prognosis. **(E)** Relationship between the disease type and risk score. **(F)** Relationship between the tumor location and risk score.

Patients with higher risk scores generally have bigger tumor sizes (T), more tumor nodes (N), and higher tumor node metastasis (M) stages (Figures 7A–C). In TCGA and GSE39582 data, the mean risk score for patients with T1, T2, T3, and T4 stages increased sequentially (Supplementary Table S2). The risk score is also related to the disease type and tumor location. The risk score is an independent prognostic factor associated with OS (*p* = 0.007), as determined by TCGA analysis (Figure 7D). Moreover, in GSE39582, the risk score also acted as a prognostic indicator of COAD (*p*< 0.001) (Figure 7G). Adenomas and adenocarcinomas had lower risk scores than mucinous and serous neoplasms (*p* = 0.0022) (Figure 7E). The risk score of patients with proximal COAD was higher than distal COAD (*p* < 0.05) (Figure 7F). Together, these results strongly demonstrated the correlation between prognostic gene sets and tumor clinical features.

### The nomogram based on the prognostic gene sets and clinical attributes

**FIGURE 8:**
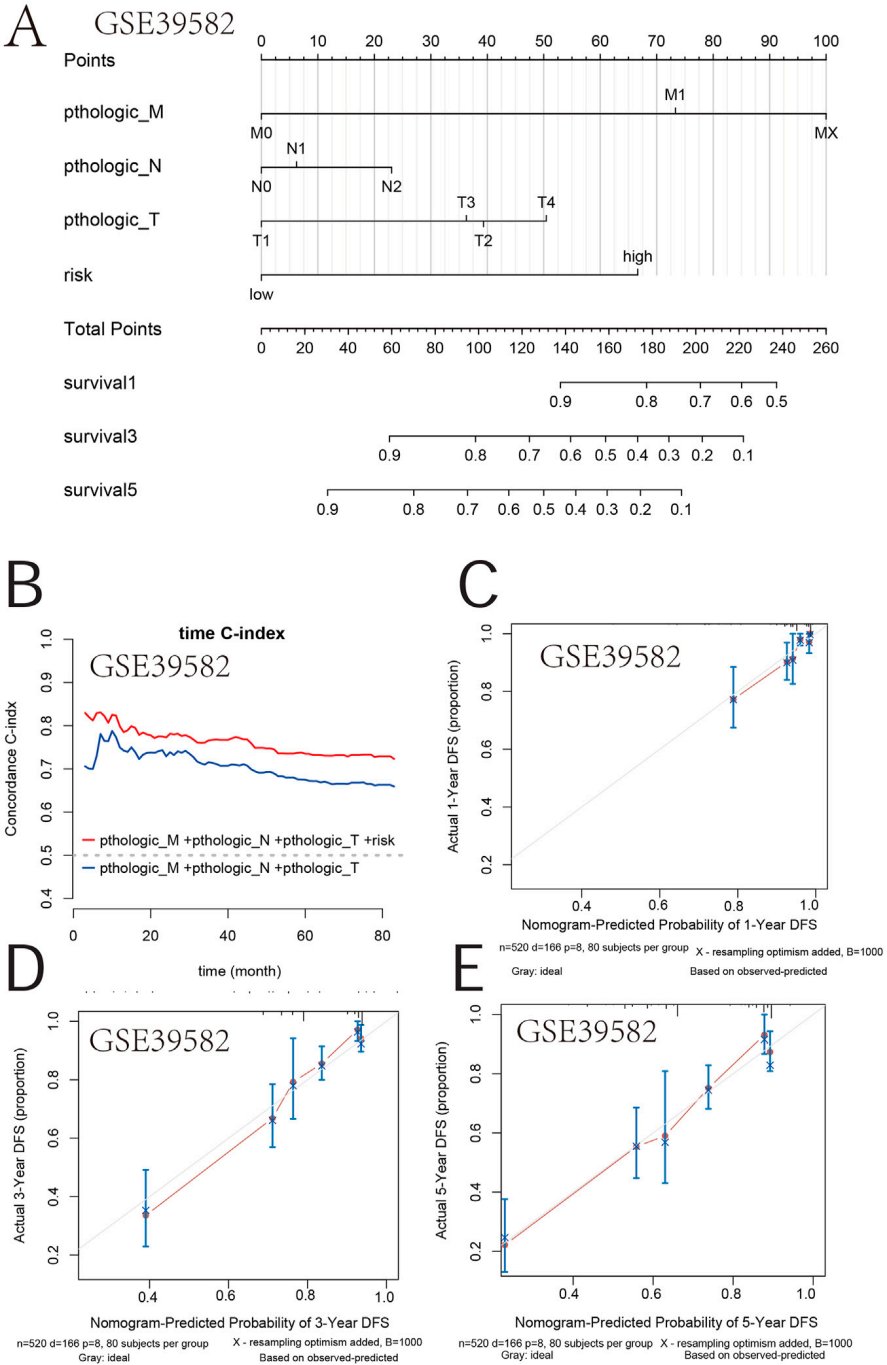
Survival nomogram. **(A)** Nomogram for the overall survival was developed in the primary cohort with three prognostic factors: pthologic M, pthologic N, and pthologic T. **(B)** Compared with the TNM and TNM + risk, the novel nomogram exhibited a better powerful capacity for survival prediction. **(C–E)** Nomogram predicting the 1-, 3-, and 5-year overall survival of COAD patients in GSE39582.

Furthermore, a nomogram integrating the genetic risk score (high risk vs. low risk) and TNM stage was constructed to provide quantitative methods to predict a patient’s probability of OS to the clinician (Figures 8A, B). The total points were calculated by adding the risk score and TNM-stage points. To evaluate the effect of the nomogram model, we also calculated its C-index. The C-index for the TNM stage with the risk score was higher than that for the TNM stage, indicating that this model is a valuable indicator for prognostic prediction (Figures 8C, E). The calibration curve for predicting a 1-, 3-, and 5-year DFS indicated that the nomogram-predicted survival closely corresponded with actual survival outcomes in GSE39582 (Figures 8D, G, H). In TCGA, the calibration curve for predicting the 1-, 3-, and 5-year DFS indicated that the nomogram-predicted survival closely corresponded with actual survival outcomes (Figures 8F, I, J). These results showed that the prognostic model accurately predicted a patient’s OS probability.

### Correlation of the prognostic gene sets with adjuvant chemotherapy

**FIGURE 9:**
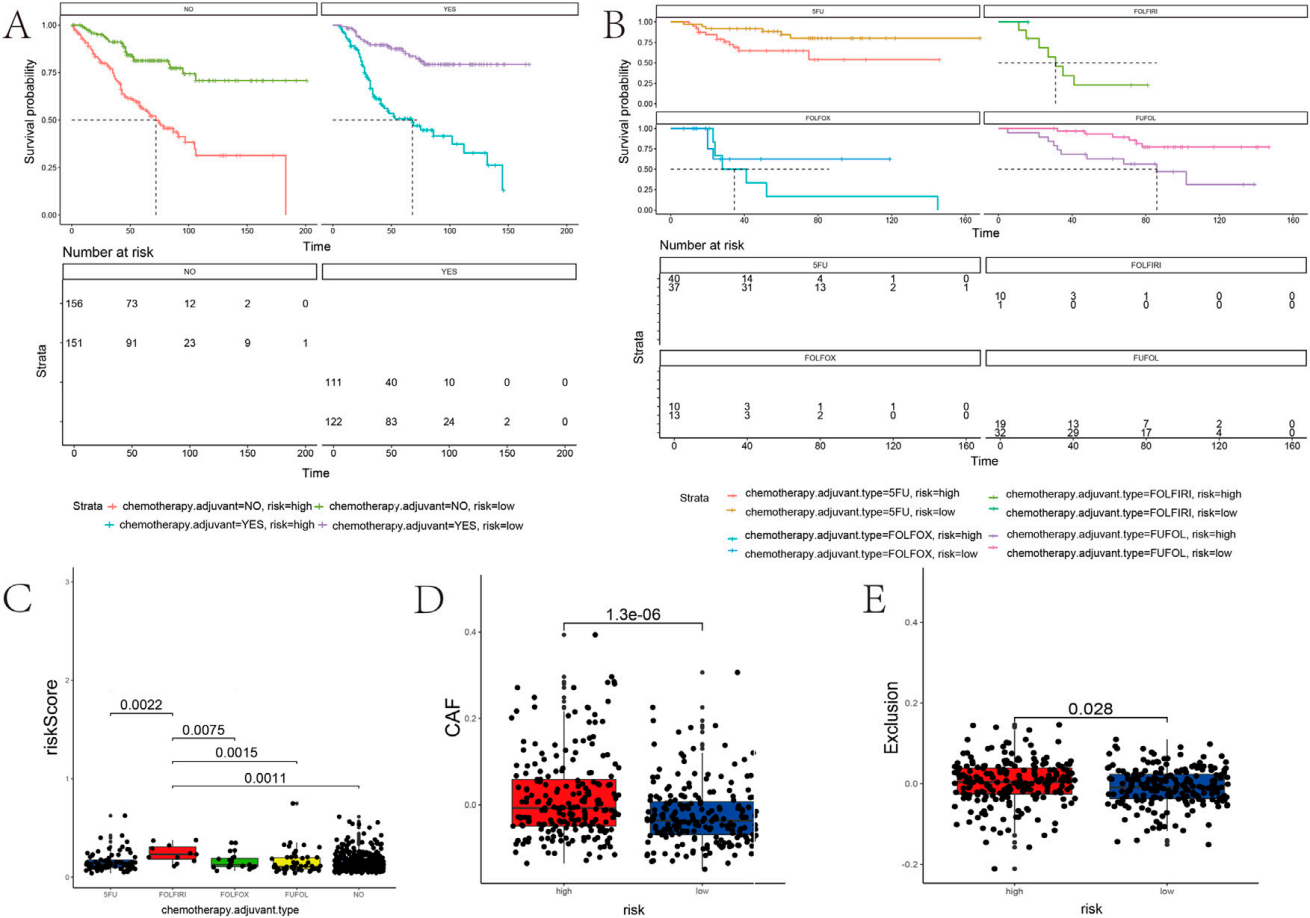
Association between the risk score and the adjuvant chemotherapy and immunotherapy response of PD-1. **(A,B)** Kaplan–Meier curves stratified by quartiles of risk scores and the usage of adjuvant chemotherapy. **(C)** Correlation of the risk score with a chemotherapy drug. **(D,E)** Risk score positively correlated with *CAF* and immune exclusion.

The survival time of patients receiving adjuvant chemotherapy was statistically significant in the high-risk group compared to the low-risk group (*p* < 0.0001), as the same in patients without adjuvant chemotherapy (Figure 9A). In addition, adjuvant chemotherapy with 5-fluorouracil, FOLFOX (folinic acid, 5-fluorouracil, and oxaliplatin), FOLFIRI (5-fluorouracil, folinic acid, and irinotecan), or FUFOL (5-fluorouracil and folinic acid) was associated with a better prognosis in both the low-risk groups than in the high-risk group (Figure 9B). The results of multiple comparative analyses of survival curves showed that patients receiving FOLFIRI chemotherapy had the worst prognosis (Figure 9C). Through further analysis, patients receiving FOLFIRI chemotherapy also had the highest risk score (Supplementary Figure S5). These results suggest that the risk score can predict the prognosis of patients treated with chemotherapy.

### Correlation of the prognostic gene sets with immunotherapy

**FIGURE 10:**
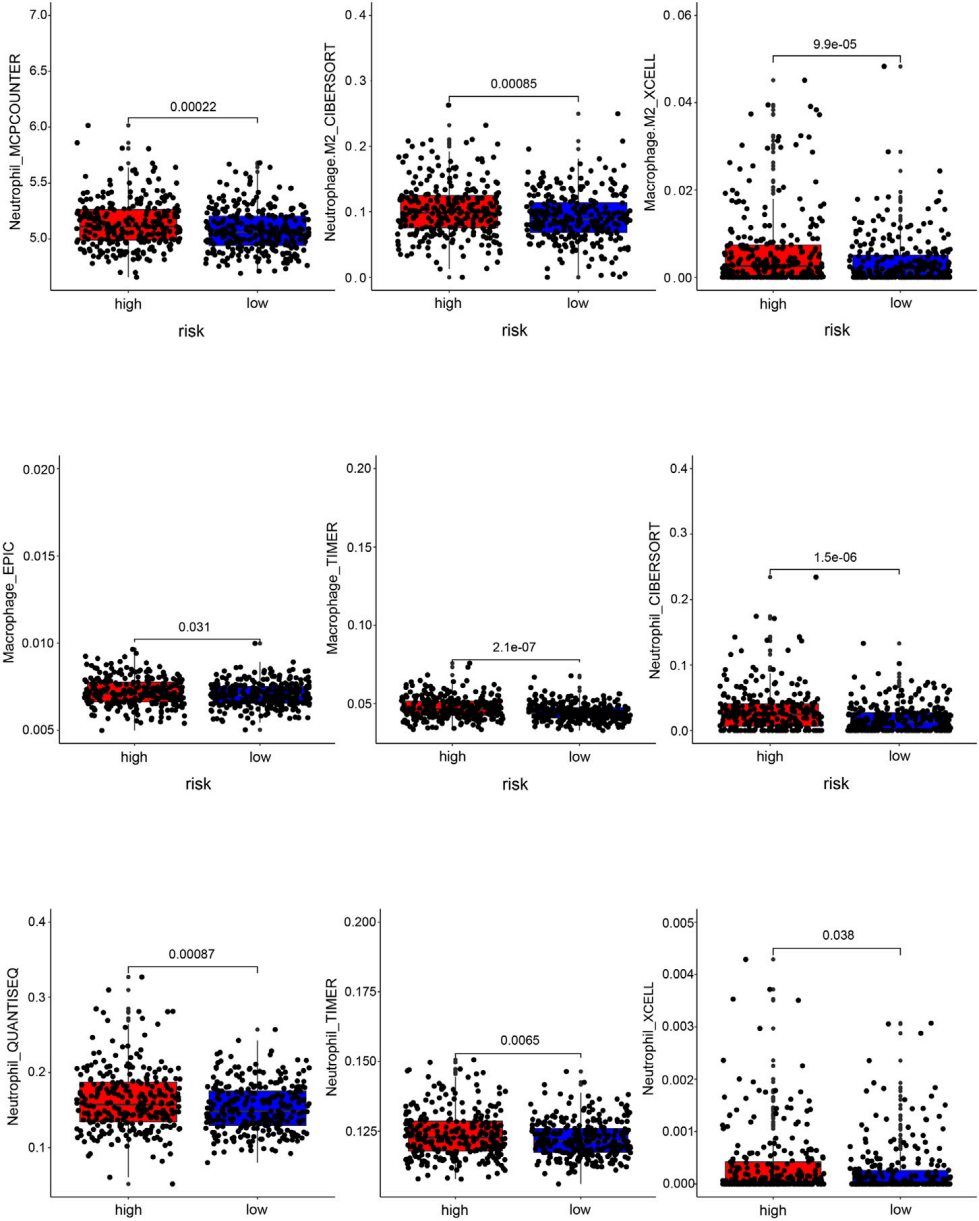
Correlation analysis between the risk score and immune cell infiltration. Box plots of immune cells with significant difference in high- and low-risk groups.

The risk score model might reflect the tumor immune microenvironment status in COAD patients, implying that the prognostic gene set also closely correlates with immunotherapy. The risk score positively correlated with the cancer-associated fibroblasts (CAF) (*p* < 0.0001) (Figure 9D). Patients with high-risk scores have a higher probability of immune exclusion than those with low-risk scores (*p* = 0.028) (Figure 9E). Subsequently, we found that the content of neutrophils and macrophages was significantly higher in a high-risk group than that in a low-risk group (*p* < 0.05) (Figure 10). These results collectively suggested that patients with a low-risk score may be better suited to undergo immunotherapy.

### Correlation between drug sensitivity and prognostic gene sets

**FIGURE 11:**
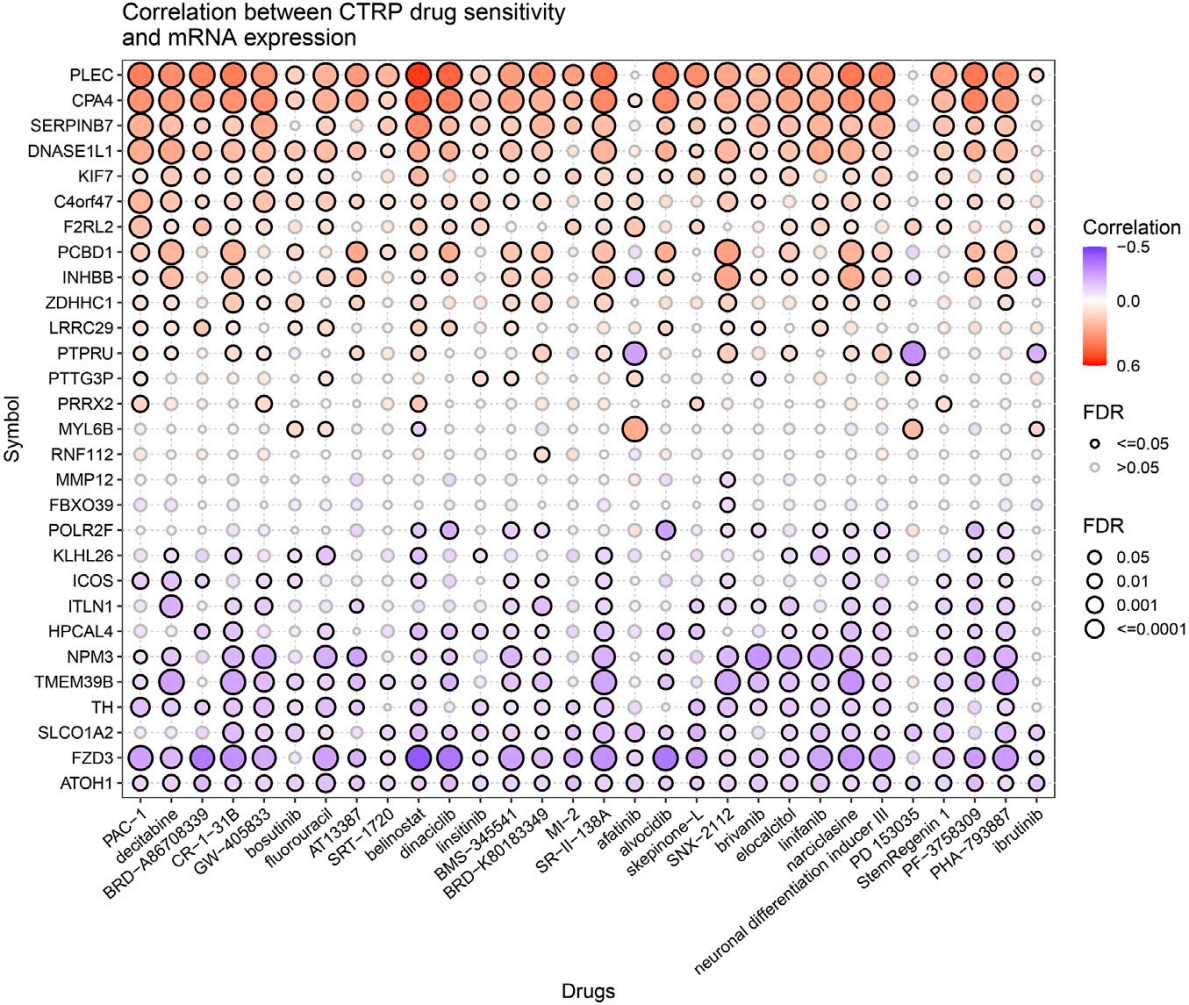
Correlation between CTRP drug sensitivity and mRNA expression.

We analyzed the correlation between drug sensitivity and predictive gene sets to further explore the value of prognostic gene sets in a clinical treatment. Most of the genes in the prognostic gene set had correlations between gene expression levels and drug sensitivity (Figure 11). The high expression levels of *POLR2F*, *KLHL26*, *ICOS*, *ITLN1*, *HPCAL4*, *NPM3*, *TMEM39B*, *TH*, *SLCO1A2*, *FZD3*, and *ATOH1* genes were resistant to drugs. The high expression of *PLEC*, *CPA4*, *SERPINB7*, *DNASE1L1*, *KIF7*, *C4orf47*, *F2RL2*, and *PCBD1* genes with an elevated expression was more sensitive to drugs. *PLEC*, *CPA4*, *SERPINB7*, *DNASE1L1*, *C4orf47*, *KIF7*, and *F2RL2* were prognostic genes positively associated with the classical antitumor drug fluorouracil. In contrast, *ATOH1*, *FZD3*, *SLCO1A2*, *TH*, *TMEM39B*, and *NPM3* were prognostic genes negatively related to fluorouracil (*p* <0.001). The expressions of *PLEC*, *CPA4*, and *SERPINB7* positively correlated with belinostat sensitivity (*p* <0.001). The expressions of *FZD3*, *TMEM39B*, and *NPM3* negatively correlated with narciclasine sensitivity (*p* <0.001). The higher the expression of *PTPRU*, the lower the drug sensitivity of afatinib and PD153035 is (*p* <0.001). Thus, the prognostic gene set is a useful clinical tool for guiding drug use.

## Discussion

COAD is a common aggressive malignant tumor, with a high mortality rate worldwide (Biller and Schrag, 2021). The etiology and pathology of COAD are highly variable within individuals. For patients with COAD, the current standard treatment includes early surgical resection, following which, patients usually would receive immunotherapy and adjunct chemotherapy, thereby improving the overall survival rate. Even then, there are still many COAD patients who suffer a relapse and would die due to disease recurrence and distant metastasis (Goldstein et al., 2014). So far, the specific underlying molecular pathogenesis of COAD remains largely unclear. Considering COAD’s poor prognosis, the need of the hour is to develop a model to predict survival outcomes of COAD patients based on prognosis risk-related gene expression profiling. Currently, COAD patients are diagnosed by the pathophysiological evaluation of prognostic molecular markers (Dekker and Rex, 2018). However, the current biomarkers of COAD are inadequate to predict patients’ survival accurately. A single biomarker may not be suitable for the treatment of every patient. Due to individual patient-specific differences, the expression of biomarkers is usually not the same. These biomarkers also fail to predict which patients will benefit from the treatments.

In this study, we used a bioinformatically developed and validated novel prognostic gene set that was significantly associated with OS in COAD patients. A risk score model was also constructed to divide COAD patients into high- and low-risk groups. The Kaplan–Meier survival analysis with the log-rank test and ROC was used to establish the prognostic ability of the model. More importantly, by establishing a validation set, we further verified the reliability of this risk score model.

Moreover, the novel prognostic gene set is closely correlated with pro-tumorigenic signatures, including the cell cycle, DNA damage, DNA repair, angiogenesis, metastasis, proliferation, differentiation, stemness, apoptosis, hypoxia, EMT, invasion, inflammation, and quiescence. Many studies showed that these hallmarks of cancer and the immune microenvironment dictate the disease prognosis in COAD. Furthermore, the correlation between the risk score model and gene mutation was also illustrated. The gene mutation probability was significantly higher in a high-risk group than in a low-risk group, which to the best of our knowledge, substantially contributes to cancer progression. Further investigations are necessary to determine the potential functional mechanisms underlying these prognosis risk-related genes. Collectively, our risk score model might be reliable in predicting the prognosis of COAD based on these results.

COAD patients treated with 5-fluorouracil, oxaliplatin, irinotecan, and folinic acid (used sequentially or together upfront) have a better objective response and survival outcome (Wang et al., 2018). 5-Fluorouracil is the most widely used drug and has a low impact on the survival rate (Golfinopoulos et al., 2007). As a result, FOLFOX, FOLFIRI, and FUFOL were in a clinical practice and substantially affected the survival rate (Harada et al., 2019). Higher toxicity renders significant side effects from chemotherapy, warranting careful evaluation before the complete use, limiting it to a small group of patients. Using this scoring model to predict the effect of chemotherapy in COAD patients, the survival advantage in the low-risk group was significant among patients who received chemotherapy. According to our analysis, chemotherapy treatments with FOLFIRI have a higher risk score than 5-fluorouracil, which may deteriorate the survival rate. The scoring model based on prognostic gene sets can efficiently predict the chemotherapy effect of COAD patients.

In addition to adjuvant chemotherapy, immunotherapy is one of the most common treatments for patients with COAD with or without metastasis. The immune microenvironment dictates the efficacy of immune drugs. Hence, patients who use the same therapy during the same phase may have different therapeutic effects. Immune infiltrating cells play essential roles in the progression of COAD (Huang et al., 2019). The prognosis of patients with COAD is mainly related to immunity. Recent studies have demonstrated that a higher density of CD4 naive T cells, regulatory T cells, and M2 macrophages is closely associated with a worse clinical prognosis in many malignant tumors, including COAD (Komohara et al., 2016; Speiser et al., 2016; Labanieh et al., 2018). In contrast, naive B cells, CD8 T cells, and CD4 memory-activated T cells were the protective factors of patients (Yang et al., 2019). Moreover, *CAF* promotes cancer progression by inducing an immunosuppressive tumor microenvironment, rendering resistance to immunotherapy (Miyai et al., 2020; Abuwarwar et al., 2021). Therefore, studying tumor immune infiltration helped analyze the patient’s prognosis and develop new cancer diagnosis and treatment methods. The risk score positively correlated with the *CAF* and immune exclusion. Therefore, immunotherapy may be less effective in patients with high-risk scores than in patients with low-risk scores—these risk scores guide immunotherapy decision-making.

Previous studies have also built prognostic models for COAD. Wang et al. developed and validated a novel stem-related prognostic model (AUC = 0.705) for COAD cancer (Wang et al., 2021). Li et al. established a COAD resistance prediction model (AUC = 0.659), which provides therapeutic targets for COAD (Li et al., 2022). The AUC values of our prognostic model at 1, 3, and 5 years in the training set were 0.73, 0.75, and 0.76, respectively, which were all higher than the aforementioned AUC values. In addition, through further verification, it is found that our prognosis model also has a medium accuracy for predicting the survival of COAD patients with 1, 3, and 5 years in the verification set, further confirming the enhanced performance of our prognostic model, hypothesizing that it may likely become a new type of COAD prognostic index. Our study also analyzed the relationship between predictive gene sets and anticancer drug susceptibility, providing novel insights into the search for selecting a more effective anticancer drug therapy and avoiding tumor resistance. Univariate and multivariate independent prognostic analyses showed that the predictive gene set and TNM stage were critical, independent predictors of COAD OS.

Furthermore, we generated a nomogram to quantify the risk assessment and survival probability. Compared to TNM, the nomogram exhibited the highest accuracy and discrimination in OS prediction. In addition, the new prognostic gene set can guide the clinical application of chemotherapy, immunotherapy, and targeted drugs. Therefore, our predictive model may help avoid unnecessary overtreatment of indolent disease and select the best management strategy. However, there are still some limitations to this study. Pure bioinformatics analysis is the main drawback of this study. Second, the interaction between genes in the prognostic gene set should be investigated better to understand the molecular mechanism of COAD occurrence and progression.

## Conclusion

Our study profiled a novel risk score model based on 33 genes for predicting the overall survival in COAD patients. More importantly, the risk score model is significantly associated with the unfavorable clinical outcome of COAD and might monitor its development to provide more effective personalized therapeutic decision-making. A nomogram model might aid in identifying high-risk COAD patients and selecting appropriate clinical follow-up plans accordingly.

## Data Availability

The original contributions presented in the study are included in the article/Supplementary Material; further inquiries can be directed to the corresponding authors.
