# Supplementary figures and images for: A novel prognostic gene set for colon adenocarcinoma relative to the tumor microenvironment, chemotherapy, and immune therapy
Source: Front Genet. 2023 Jan 9;13:975404. doi: 10.3389/fgene.2022.975404 (PMC9868701; doi:10.3389/fgene.2022.975404)

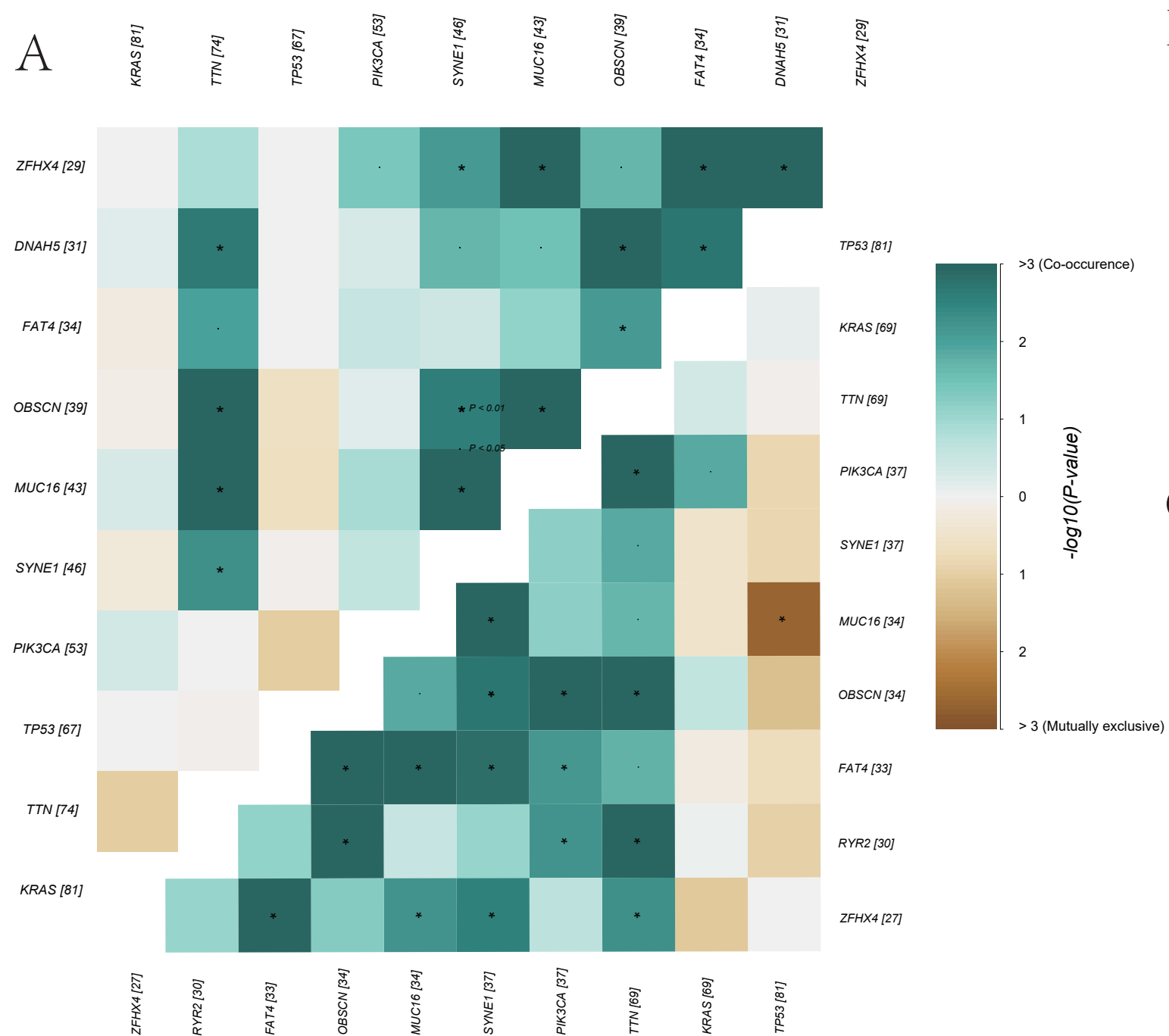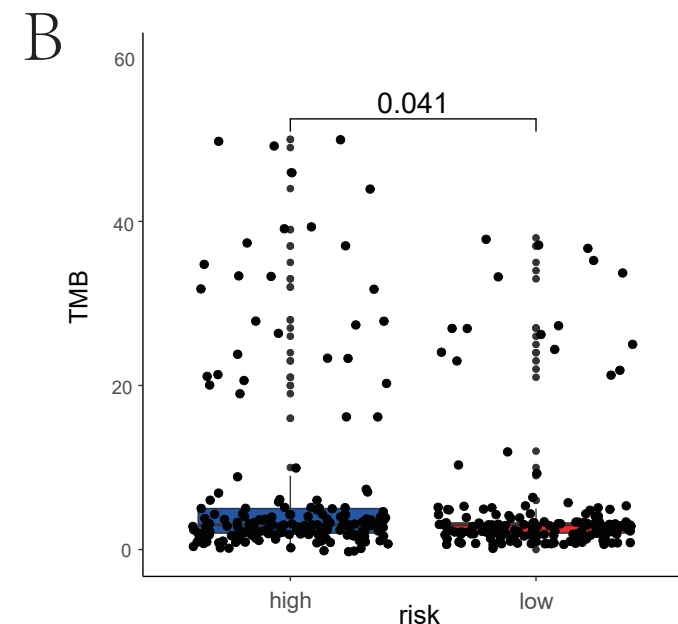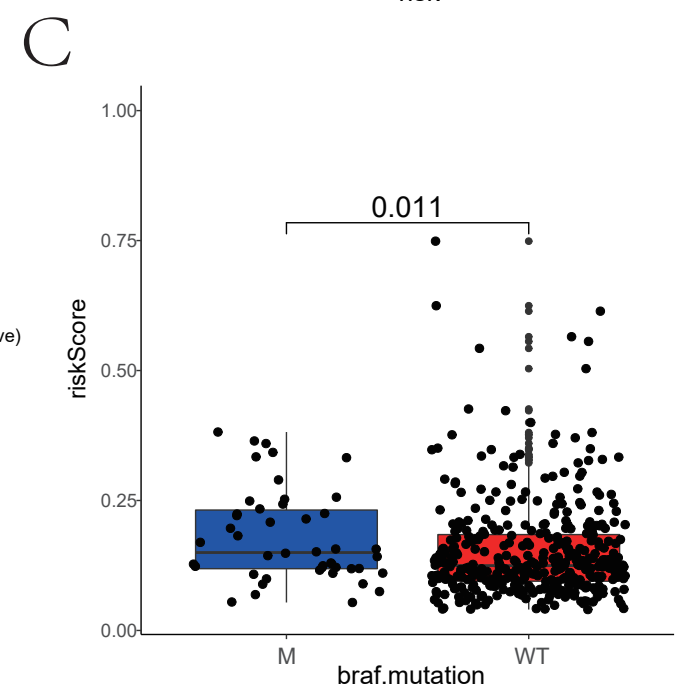

Supplement: Supplementary file 1 [file DataSheet2.PDF]

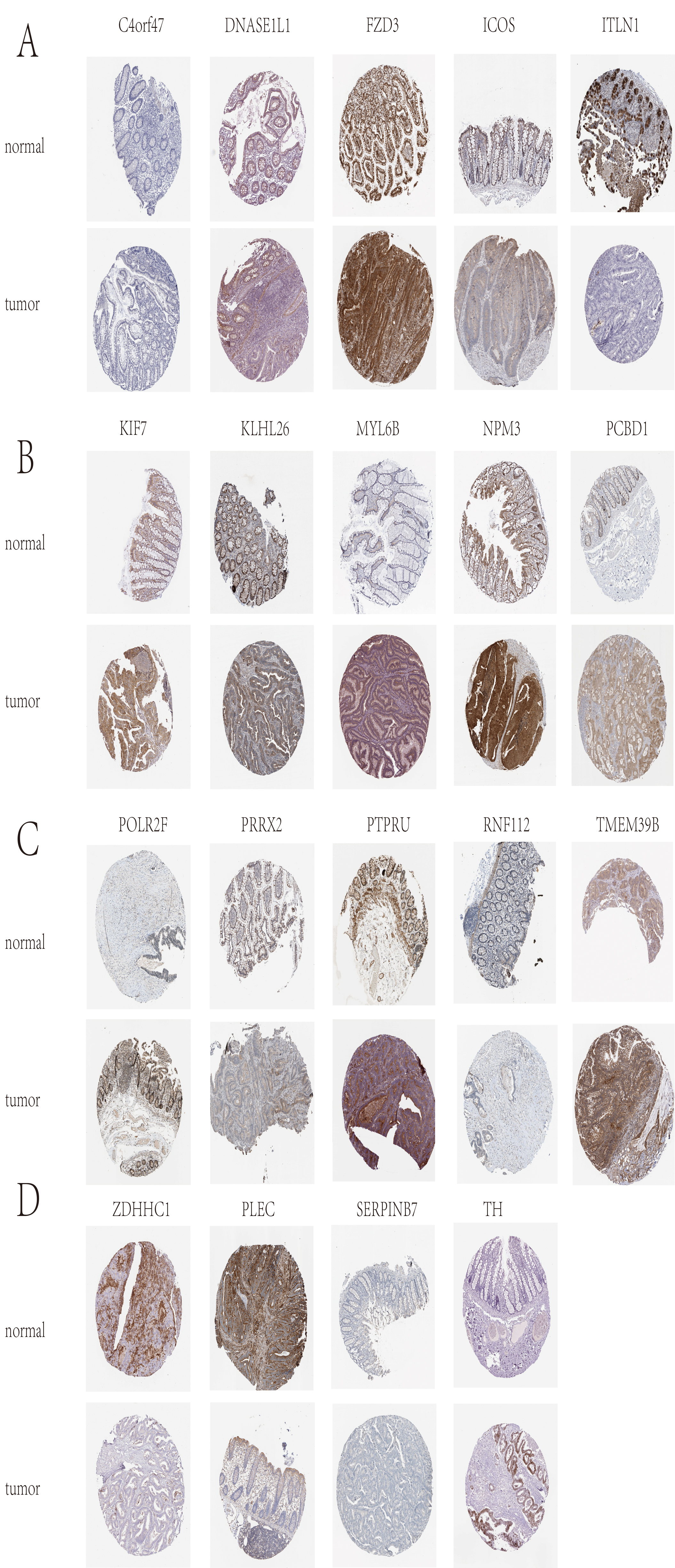

Supplement: Supplementary file 4 [file Image1.JPEG]

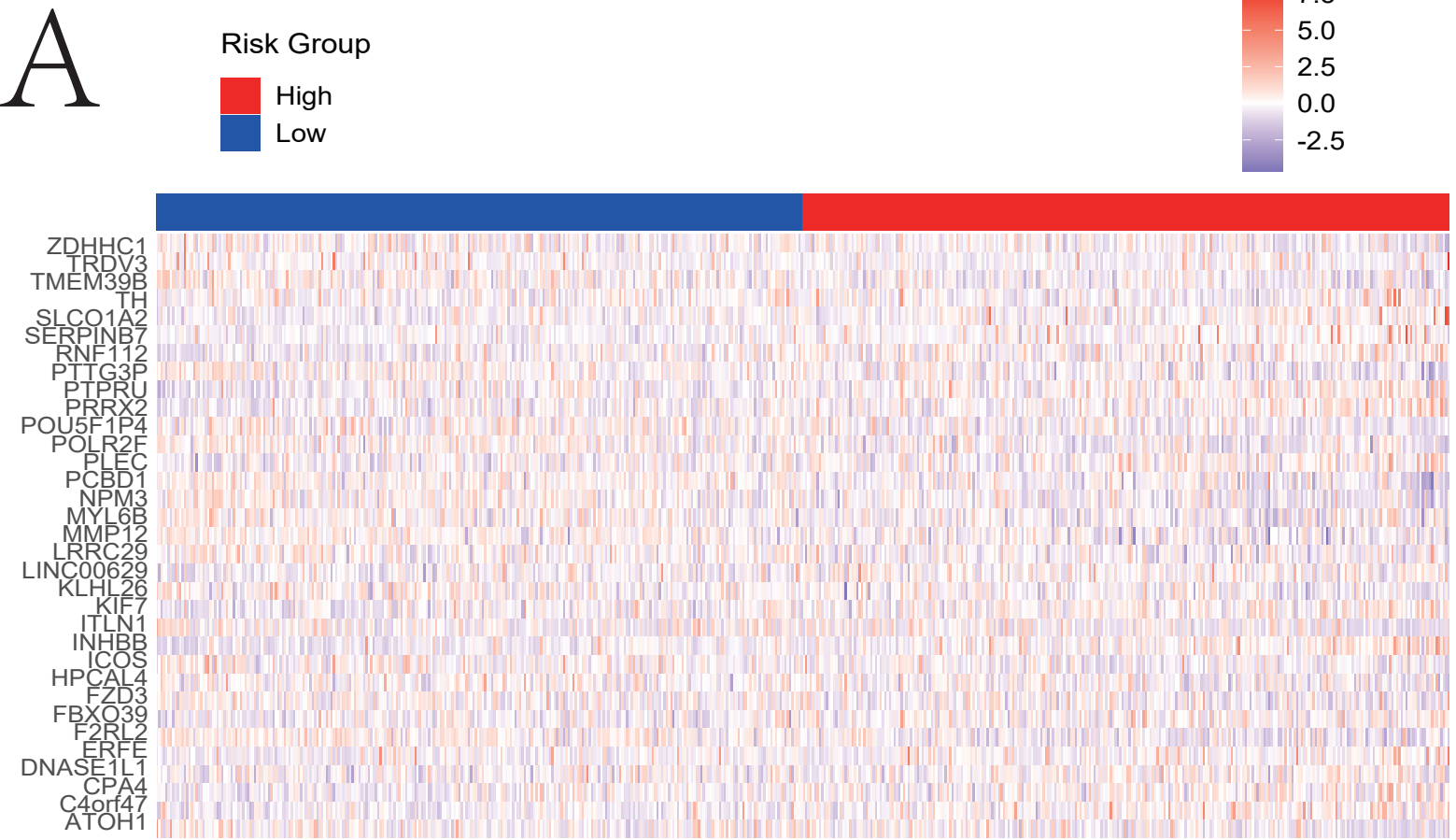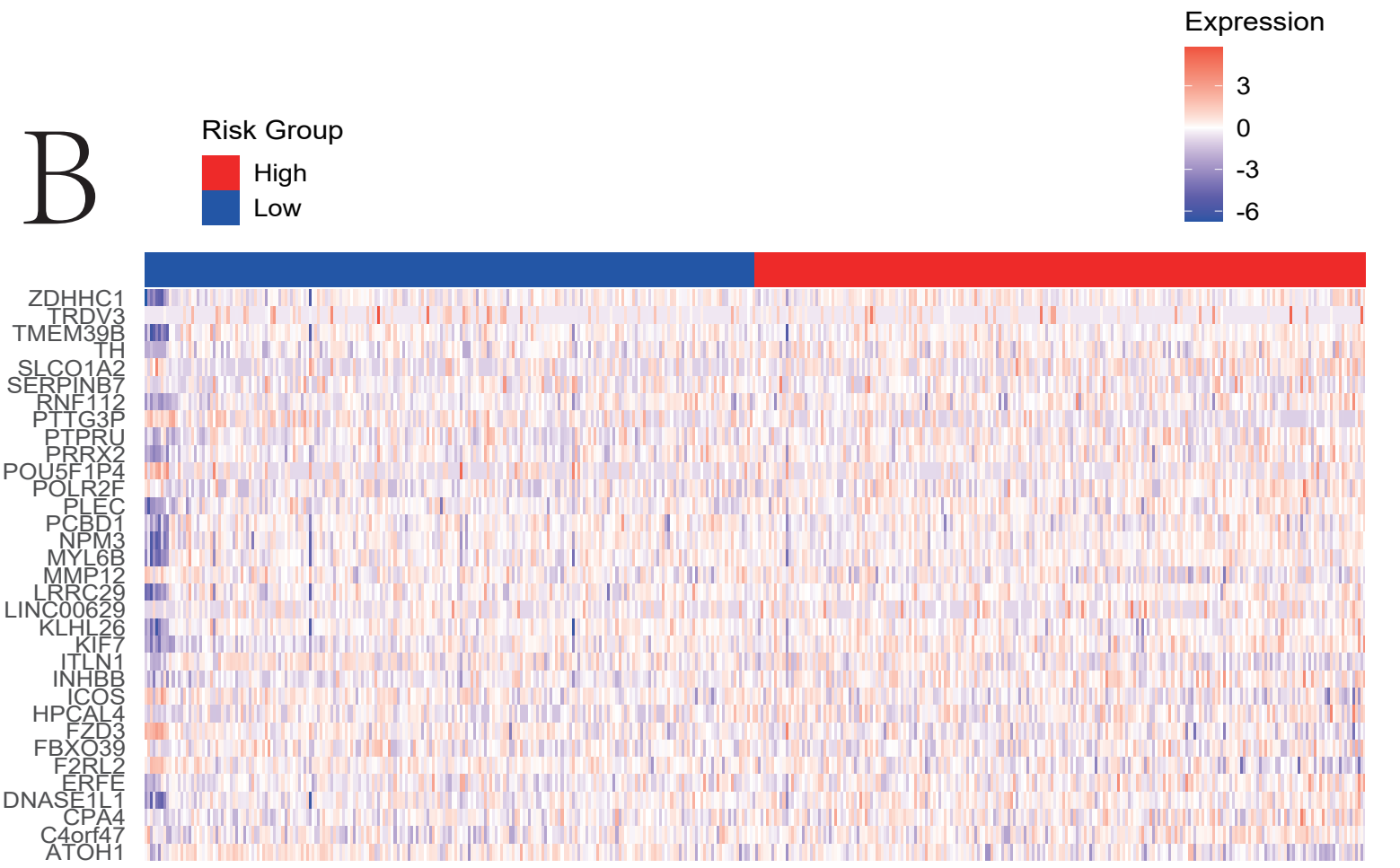

Supplement: Supplementary file 5 [file DataSheet3.PDF]

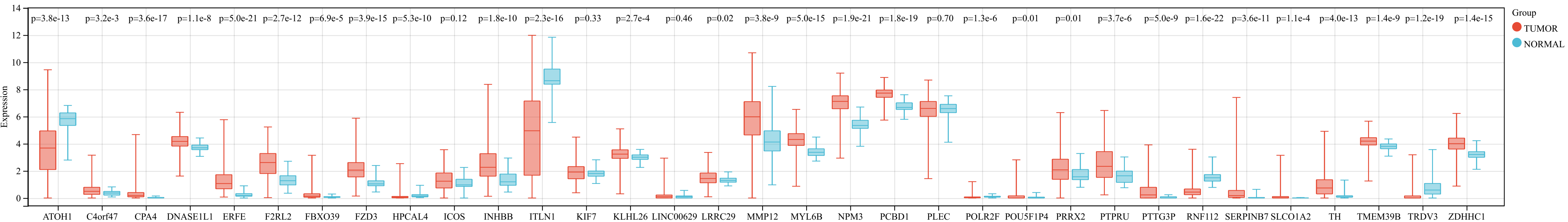

Supplement: Supplementary file 6 [file DataSheet1.PDF]
