# Supplementary material for: A novel prognostic gene set for colon adenocarcinoma relative to the tumor microenvironment, chemotherapy, and immune therapy
Source: Front Genet. 2023 Jan 9;13:975404. doi: 10.3389/fgene.2022.975404 (PMC9868701; doi:10.3389/fgene.2022.975404)
Supplement: Supplementary file 3 [file DataSheet4.PDF]

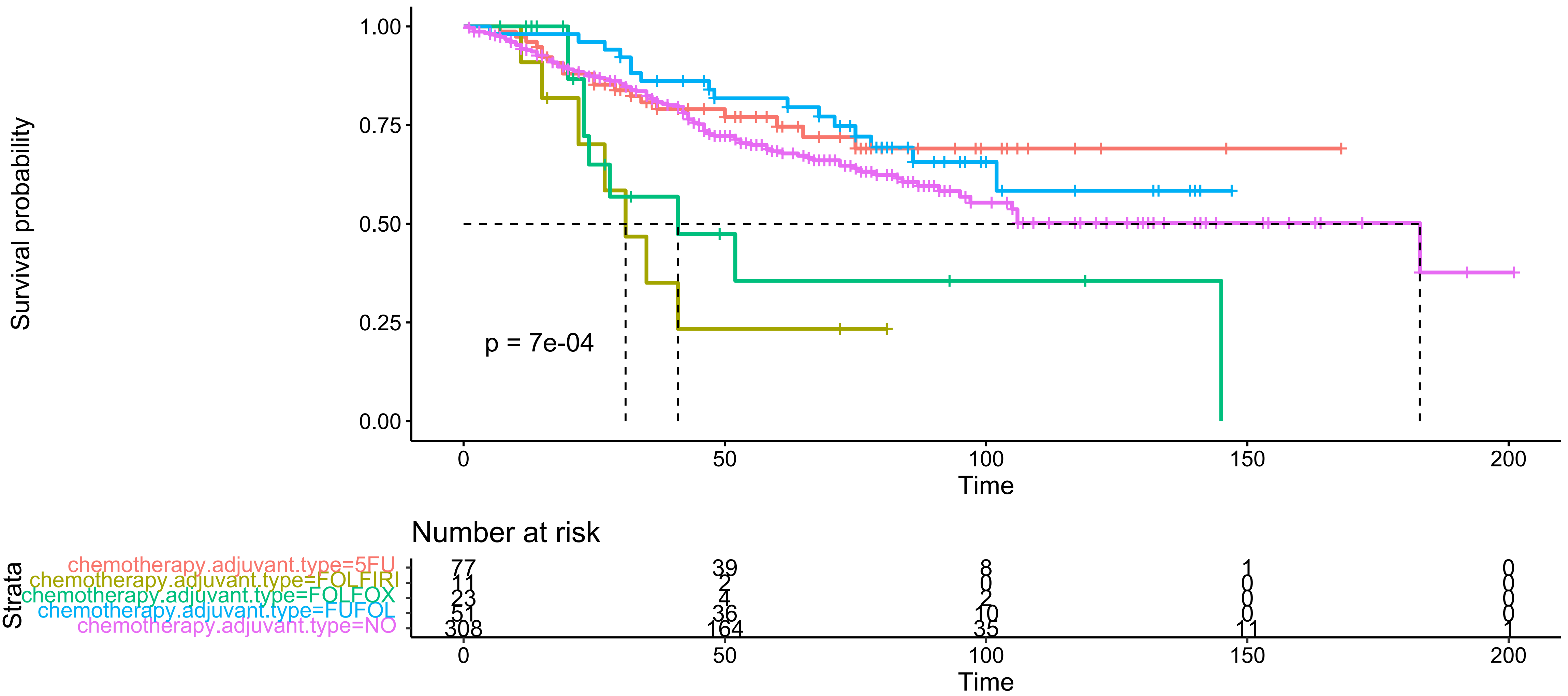
Survival probability
1.00
0.75
0.50
0.25
0.00
p = 7e-04
0
50
100
150
200
Time
Number at risk
Strata
chemotherapy.adjuvant.type=5FU 77 39 8 1 0
chemotherapy.adjuvant.type=FOLFIRI 11 2 0 0 0
chemotherapy.adjuvant.type=FOLFOX 23 4 2 0 0
chemotherapy.adjuvant.type=FUFOL 51 36 10 0 0
chemotherapy.adjuvant.type=NO 308 164 35 11 1
0
50
100
150
200
Time
